# The Crucial Role of Tinzaparin in Managing Venous Thromboembolism in the Cancer Population

**DOI:** 10.3390/jcm14196695

**Published:** 2025-09-23

**Authors:** Alfredo Mauriello, Adriana Correra, Anna Chiara Maratea, Celeste Fonderico, Arianna Amata, Vincenzo Quagliariello, Vincenzo Russo, Antonello D’Andrea, Nicola Maurea

**Affiliations:** 1 S.C. Cardiology, Institute National Cancer, IRCCS, Foundation “G. Pascale”, 80131 Naples, Italy; annachiara.maratea@gmail.com (A.C.M.); celeste.fonderico@istitutotumori.na.it (C.F.); nicolamaurea@istitutotumori.na.it (N.M.); 2 Cardiology Department, Ospedali Riuniti University Hospital, Viale Pinto 1, 71122 Foggia, Italy; adrianacorrera@gmail.com; 3 Department of Cardiovascular Medicine, Fondazione Policlinico Universitario A. Gemelli, IRCCS, 00168 Rome, Italy; arianna.amata2@gmail.com; 4 Cardiology Unit, Department of Medical and Translational Sciences, “Monaldi Hospital”, 80131 Naples, Italy; 5 Cardiology and Intensive Care Unit, Department of Cardiology, Umberto I Hospital, 84014 Nocera Inferiore, Italy

**Keywords:** tinzaparin, heparin, cancer, anticoagulation, thromboembolism, bleeding

## Abstract

**Background:** Venous thromboembolism (VTE) is a serious and common complication in cancer patients, and it is the second leading cause of death after cancer itself. Cancer-associated thrombosis (CAT) is an indicator of a poorer prognosis and can lead to treatment delays and increased healthcare costs. This review aims to provide a comprehensive update on the efficacy and safety of tinzaparin in the treatment and prophylaxis of VTE in cancer patients. **Methods:** This is a narrative review that examines the pharmacological properties of tinzaparin, as well as the results from clinical studies and meta-analyses. It includes a discussion of tinzaparin’s role in special patient populations and its comparison with other anticoagulants. **Results:** Tinzaparin is a low-molecular-weight heparin (LMWH) that does not accumulate in patients with renal insufficiency, eliminating the need for dose adjustments. Studies have shown that tinzaparin is a safe and effective treatment for CAT, with a favorable safety profile regarding hemorrhagic complications. In the CATCH study, tinzaparin showed a significant reduction in clinically relevant non-major bleeding compared to warfarin. Tinzaparin has also been shown to be more effective than vitamin K antagonists (VKAs) in promoting the recanalization of venous thrombi. A meta-analysis confirmed that tinzaparin was superior to VKAs in preventing VTE recurrence in the long term. **Conclusions:** While direct oral anticoagulants (DOACs) offer convenience, LMWHs like tinzaparin remain crucial, especially for patients with specific characteristics such as renal insufficiency, complex drug interactions, or a high risk of gastrointestinal bleeding. Tinzaparin’s favorable safety and efficacy, along with its unique pharmacological properties, make it a valuable option for managing VTE in the complex oncology population.

## 1. Introduction

**Figure 1:**
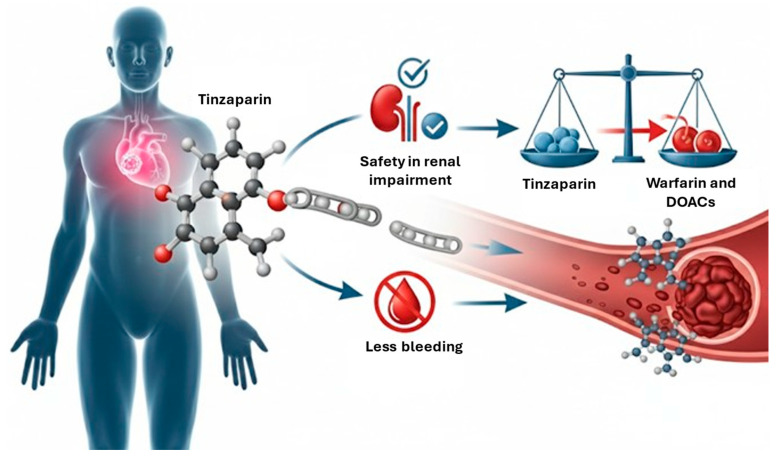
Graphical abstract. Red arrow indicates that tinzaparin is superior to warfarin and DOACs in patients with renal impairment. DOACs: direct oral anticoagulants.

Venous thromboembolism (VTE), which covers deep vein thrombosis (DVT) and pulmonary embolism (PE), is a severe and common complication in cancer patients, and it is the second leading cause of death after cancer itself [1]. Cancer patients have a four- to seven-fold higher risk of VTE compared to the general population [2]. This increased incidence is attributed to increased patient survival, more aggressive anticancer treatments, and better awareness of cancer-associated thrombosis [3]. Cancer-associated thrombosis (CAT) is an indicator of poorer prognosis and contributes significantly to morbidity, delays or interruptions in chemotherapy, and increased healthcare costs [4]. The risk of CAT varies considerably depending on the type and stage of cancer, with pancreatic, brain, lung, ovarian, and multiple myeloma cancers associated with the highest risks. Furthermore, locally advanced and metastatic cancers carry a greater VTE risk than localized stages [5]. Managing CAT is complex due to the high propensity of patients for both recurrent thrombotic events and hemorrhagic complications [6]. Cancer patients undergoing anticoagulant therapy often receive combined anticancer therapies and suffer from multiple comorbidities such as renal insufficiency, thrombocytopenia, or brain metastases, making the choice and management of the anticoagulant particularly challenging [7,8]. For many years, low-molecular-weight heparins (LMWHs) have been the standard of care for the long-term treatment of CAT, based on the results of randomized controlled trials (RCTs) that demonstrated the superiority of LMWH monotherapy over “bridged” vitamin K antagonist (VKA) therapy, with a similar or superior safety profile [7]. Among the available LMWHs, tinzaparin stands out for a series of theoretical and practical advantages. It is authorized for the treatment of CAT in most European Union countries, with the exception of Austria and Italy [9]. Tinzaparin has more stable pharmacokinetic properties and fewer drug interactions than VKAs. It is administered at a full therapeutic dose (175 anti-Xa IU per kg of body weight) once daily and does not require dose adjustments over time [1]. Unlike other LMWHs, tinzaparin is a particularly attractive option, because it does not accumulate in patients with renal insufficiency (RI), a frequent comorbidity in cancer patients. It also shows the highest rates of anti-Xa activity reversal in response to protamine sulfate among all LMWHs [10]. However, the use of tinzaparin also has several critical points. Specifically, there is still no comparative data on its efficacy and safety compared to direct oral anticoagulants (DOACs), and there is a lack of clarity regarding its long-term use. The aim of this comprehensive review is to provide a comprehensive update on the efficacy and safety of tinzaparin in the treatment and prophylaxis of VTE in cancer patients, based on the most recent scientific literature and clinical guidelines. We will discuss its pharmacological properties, the results of clinical studies in different cancer patient populations, and a comparison with other anticoagulants. Figure 1 represents a graphical abstract.

## 2. Pharmacological Profile of Tinzaparin

Tinzaparin is an LMWH derived from the enzymatic depolymerization of unfractionated heparin (UFH) of porcine origin [11]. Its anticoagulant action works by enhancing the inhibitory effect of antithrombin on coagulation factors, particularly Factor Xa and Factor IIa [12]. Tinzaparin is characterized by the lowest anti-Xa/anti-IIa ratio among all LMWHs (between 1.5 and 2.5 times the normal ratio) and the highest release of tissue factor pathway inhibitor (TFPI), a potent coagulation inhibitor. Plasma anti-Xa activity is considered the biomarker for LMWH activity [12]. Tinzaparin also has the highest molecular weight among LMWHs [12].

At the pharmacokinetic level, tinzaparin follows first-order kinetics, with elimination occurring primarily through cellular and hepatic pathways and, to a lesser extent, through renal clearance (RC) [13]. This profile is particularly advantageous in patients with RI because bioaccumulation does not occur even in cases of severe RI, eliminating the need for dose adjustments [13]. The elimination half-life of tinzaparin is 3.4–4.1 h after subcutaneous injection and 1.6 h after intravenous administration [13]. Tinzaparin administration does not influence hemoglobin levels or platelet count [13].

Another distinctive feature of tinzaparin is its high rate of anti-Xa activity reversal in response to protamine sulfate [14]. These properties give tinzaparin a favorable safety profile, with a lower risk of bleeding compared to other anticoagulants. In addition, tinzaparin is administered at a full therapeutic dose (175 anti-Xa IU per kg of body weight) once daily, without the need for dose reductions over time. This aspect, combined with its safety in the elderly and patients with RI, makes it a practical and efficient option, especially in a complex population like cancer patients, who may require indefinite anticoagulant therapy [10]. Table 1 summarizes the different pharmacokinetic profile among several LMWHs and DOACs.

## 3. Tinzaparin in the Prevention and Treatment of Cancer-Associated Thrombosis

The efficacy and safety of tinzaparin for the prevention and long-term treatment of DVT and PE in patients with active cancer have been the subject of numerous clinical studies, including randomized trials and meta-analyses. These studies have compared tinzaparin with VKAs and, more recently, with other LMWHs, providing essential data on its role in the management of CAT.

### 3.1. Clinical Trials About Efficacy of Tinzaparin

In the CLOT study [19], a multicenter, randomized, open-label clinical trial, including 672 cancer patients with acute venous thromboembolism, dalteparin was more effective than warfarin in reducing the risk of recurrent thromboembolism [hazard ratio (HR), 0.48; *p* = 0.002] without increasing the risk of bleeding during a follow-up of six months.

The LITE trial [20], a multicenter, open-label RCT, randomized 200 CAT patients to receive either tinzaparin or warfarin for 3 months. During a follow up of 12 months, the DVT recurrence rate was 7% in the tinzaparin group and 16% in the warfarin group [relative risk (RR) = 0.44; absolute difference −9.0; 95% confidence interval (CI) from −21.7% to −0.7%; *p* = 0.07]. Despite the small sample size, these results were consistent with the CLOT study. No difference in the increase in the overall bleeding risk was found with tinzaparin (27% vs. 24% in the warfarin group; *p* = 0.77), and mortality was similar in both arms (47%; *p* = 0.54).

The Main-LITE Trial [21], a multicenter, open-label RCT on 200 VTE cancer patients compared long-term therapeutic tinzaparin subcutaneously once daily with usual-care long-term VKAs therapy for 3 months. Of 200 patients, 100 received tinzaparin and 100 received usual care. At 12 months, the usual-care group had an excess of recurrent VTE; 16 of 100 (16%) versus 7 of 100 (7%) receiving LMWHs (*p* = 0.044; risk ratio = 0.44; absolute difference −9.0; 95% CI, −21.7 to −0.7). Bleeding, largely minor, occurred in 27 patients (27%) receiving tinzaparin and 24 patients (24%) receiving usual care (absolute difference −3.0; 95% CI, −9.1 to 15.1). In patients without additional risk factors for bleeding at the time of randomization, major bleeding occurred in 0 of 51 patients (0%) receiving tinzaparin and 1 of 48 patients (2.1%) receiving usual care. Mortality at 1 year was high, reflecting the severity of the cancers; 47% in each group died. This study concluded that tinzaparin was non-inferior to VKAs in the treatment of VTE and was also safer in some subset of patients, providing greater patient autonomy and reduced dependence on healthcare resources.

The Home-LITE Trial [22], a multicenter, open-label RCT, divided 480 patients with acute DVT (25% with cancer) into a tinzaparin arm and a warfarin arm to compare long-term home treatment. VTE recurrence rates were 3.3% in both groups during the 12-week study period and also at the 1-year follow-up (10.4% vs. 8.3% in the tinzaparin/VKAs groups, respectively). Mortality and bleeding rates were similar. Perceived treatment satisfaction was significantly higher in the tinzaparin arm (*p* = 0.024), mainly due to the lack of interference with daily activities. A retrospective analysis of the Home-LITE Trial [22] showed the superiority of tinzaparin (overall odds ratio OR: 0.76, *p* = 0.004) in significantly reducing the incidence of venous ulcers and post-thrombotic syndrome (PTS), a frequent and costly sequela of VTE. The most significant benefit was found in patients with iliac vein thrombosis (OR: 0.53).

The Romera trial [1] was an open-label randomized study that included 241 patients with symptomatic proximal lower limb DVT. Patients were treated for 6 months with full therapeutic dose tinzaparin or acenocoumarol. During the 12-month period, six patients (5%) in the tinzaparin group and 13 (10.7%) in the VKA group had a VTE recurrence (*p* = 0.11). In cancer patients, VTE recurrence tended to be lower in the tinzaparin group (two out of 36 [5.5%]) compared to the VKAs group (seven out of 33 [21.2%]; *p* = 0.06). These percentages reflect the results of the multicenter, open-label RCT on 241 patients, where after 6 months, VTE recurrence in cancer patients was 5.5% in the tinzaparin arm and 9.1% in the acenocumarol arm (*p* = 0.58), while at 12 months it was 5.5% in the tinzaparin arm and 21.2% in the acenocumarol arm (*p* = 0.06).

The CATCH study was the largest RCT [23], including 900 patients, comparing the efficacy and safety of tinzaparin for 6 months with conventional therapy using tinzaparin followed by warfarin (target INR 2.0–3.0 for 6 months) in patients with active cancer and acute symptomatic VTE. The primary efficacy outcome was a composite of recurrent DVT, fatal or non-fatal PE, and incidental VTE. Although tinzaparin did not significantly reduce the primary composite outcome of recurrent VTE (7.2% for tinzaparin vs. 10.5% for warfarin; HR 0.65; 95% CI 0.41–1.03; *p* = 0.07), it did reduce the risk of clinically relevant non-major bleeding (CRNMB). In the per-protocol analysis, the cumulative incidence of recurrent VTE was 8.3% in the tinzaparin group compared to 12.7% in the warfarin group (HR 0.62; 95% CI 0.38–1.00; *p* = 0.05). The study demonstrated that tinzaparin, even when used at the full therapeutic dose for 6 months, is safe in a broad population of cancer patients.

The TROPIQUE study [24] was a multicenter prospective observational study, including 409 cancer patients and acute symptomatic VTE. A post hoc analysis [9] on 301 cancer patients who received long-term tinzaparin treatment for a first VTE event confirmed the favorable benefit-risk ratio of tinzaparin for the long-term treatment of CAT. The cumulative VTE recurrence rate at 6 months was 5.4% (95% CI: 3.2–9.2%; *p* = 0.002). Clinical outcomes varied across cancer types, with the highest risk in patients with lung and gastrointestinal cancers. Therefore, overall adherence to clinical practice guidelines for tinzaparin prescribing was high (72.8%).

The TiCAT study [25], a prospective single-arm trial, evaluated the safety and efficacy of tinzaparin in 247 CAT patients for a 12-month treatment period. The recurrence rate was 4.5% in the first 6 months and 1.1% in the 7–12-month period. These data support the use of tinzaparin as a safe drug for the prolonged treatment of CAT beyond 6 and up to 12 months, as a low rate of recurrent VTE and major bleeding has been demonstrated.

The USCAT study [26], a retrospective non-interventional study on 432 CAT patients, described long-term follow-up with tinzaparin from the 6th to the 12th month after the index VTE event. Between 6 and 12 months, 5.7% of patients experienced a VTE recurrence and 5.1% had clinically relevant bleeding. VTE recurrence was more frequent in lung (14.3%) and colorectal cancer (6.0%), while major bleeding was more frequent in colorectal cancer (6.0%). This study, in the absence of solid data from RCTs, provided useful indications for the long-term use of tinzaparin in CAT.

Cotter et al. [27] in their retrospective observational cohort study, including 61 patients, showed no statistically significant differences between the apixaban and tinzaparin groups in terms of major bleeding events (4.8% vs. 0%, *p* = 0.33) and clinically relevant non-major bleeding events (9.5% vs. 0%, *p* = 0.1).

Based on the provided information, you can state that tinzaparin is generally considered non-inferior to VKAs and shows a possible trend toward reduced recurrence of VTE in certain patient populations, particularly those with cancer. However, the evidence is less clear regarding its superiority over other LMWHs like enoxaparin or dalteparin. Direct comparative studies between tinzaparin and these other LMWHs are limited. Table 2 summarizes several trials about efficacy of tinzaparin.

### 3.2. Clinical Trials About Efficacy of Tinzaparin in Vessel Recanalization

The Romera trial [1] showed that tinzaparin is more effective than VKAs in promoting the recanalization of venous thrombi in the lower limbs. Differences in complete clot resolution were observed after 6 months (73.1% vs. 47.5%) and 12 months (91.5% vs. 69.2%) between the tinzaparin and VKA groups, respectively (*p* < 0.001). This study also demonstrated that tinzaparin has a better ability to reopen thrombosed veins compared to acenocoumarol, as evidenced by ultrasound.

Another study, the Daskalopoulos study [28], a prospective, open-label RCT, including 108 patients, examined tinzaparin versus acenocoumarol for the six months of treatment of VTE. It revealed that thrombus lysis occurred significantly earlier and more extensively, starting at 3 months (*p* < 0.2) with tinzaparin.

However, also in this case there is a lack of robust evidence about the role of tinzaparin in vessel recanalization.

### 3.3. Clinical Trials About the Safety of Tinzaparin

Tinzaparin has shown a favorable safety profile concerning hemorrhagic complications.

In the Romera trial [1], only one major bleeding event occurred in the tinzaparin group, and three occurred in the VKA group. Three out of 122 patients in the VKA group (2.5%) and one out of 119 patients who received tinzaparin (0.8%) had a major bleed (*p* = 0.6), with no fatal hemorrhagic events.

In the CATCH study [20], major bleeding events occurred in 12 patients assigned to tinzaparin and 11 patients assigned to warfarin (HR 0.89; 95% CI 0.40–1.99; *p* = 0.77). A significant reduction in clinically relevant non-major bleeding (CRNMB) was observed with tinzaparin (49 out of 449 patients for tinzaparin vs. 69 out of 451 patients for warfarin; HR 0.58; 95% CI 0.40–0.84; *p* = 0.004). No differences in the cause of death were noted between the treatment groups, with cancer progression being the most frequent cause (69%).

In the TROPIQUE study [9], the cumulative incidence of major bleeding at 6 months was 5.8% (95% CI: 3.6–9.6%). Although not significant, the incidence of major bleeding tended to differ by cancer type, with higher rates in hematological (10.41%), lung (9.98%), and other cancer types (8.02%) compared to gastrointestinal (2.13%) or breast cancers (0%).

However, tinzaparin as other LMWHs, is associated with some side effects. In addition to bleeding, among the most dangerous side effects are the induction of osteoporosis and heparin-induced thrombocytopenia (HIT).

Gajic-Veljanoski et al. [29] conducted a meta-analysis including 14 studies: 10 clinical trials (*n*  =  4865 participants) and four observational cohort studies (3 prospective, *n*  =  221; 1 retrospective, *n * =  30). In participants with venous thromboembolism and underlying cardiovascular disease or cancer (5 RCTs, *n*  =  2280), LMWH for 3–6 months did not increase the relative risk of all fractures at 6–12 months compared to unfractionated heparin, oral vitamin K antagonists or placebo [RR = 0.58, 95% CI: 0.23–1.43; I^2^  =  12.5%]. No statistically significant increase in the risk of fractures at 6–12 months was found for cancer patients (RR  =  1.08, 95% CI: 0.31–3.75; I^2^  =  4.4%). Based on the data from two prospective cohort studies (*n*  =  166), LMWH for 3–24 months decreased mean BMD by 2.8–4.8% (depending on the BMD site) compared to mean BMD decreases of 1.2–2.5% with oral VKAs.

Food and drug Administration Adverse Event Reporting System (FAERS) database [30] case report analysis indicated that a total of 43 patients showed evidence of LMWH-induced thrombocytopenia with a median onset time of 8 days. Almost half of the events were caused by enoxaparin. The conclusion was that LMWH-induced thrombocytopenia is rare but serious, with increased risk in patients with diabetes mellitus or a surgical history.

### 3.4. Comparison with Vitamin K Antagonists

A meta-analysis by Martínez-Zapata et al. [31] included 3 RCTs (CATCH, LITE, and Romera), for a total of 1169 cancer patients, comparing tinzaparin with VKAs for the long-term treatment of CAT for at least 3 months. This analysis showed a reduction in the risk of recurrent VTE at the end of treatment (RR 0.67; 95% CI 0.46–0.99; *p* = 0.91) and at longer follow-up (RR 0.58; 95% CI 0.39–0.88; I^2^ = 6%) with tinzaparin. A lower risk of clinically relevant non-major bleeding was observed at the end of treatment with tinzaparin (RR 0.71; 95% CI 0.51–1.00; *p* = 0.05). No significant differences were found between treatments for all-cause mortality (RR 1.09; 95% CI 0.91–1.30; I^2^ = 0%) or for fatal and non-fatal major bleeding events (RR 1.06; 95% CI 0.56–1.99; I^2^ = 0%). The overall quality of evidence was considered moderate, mainly due to the small sample size in two studies and the limited number of events in the meta-analyses. This study concluded that short- and long-term treatments with tinzaparin were superior to VKAs for preventing VTE recurrence.

The meta-analysis by Laporte et al. [32] evaluated 5 open-label RCTs to investigate tinzaparin versus VKAs in the long-term treatment of VTE. In cancer patients, the meta-analysis (1668 patients, 24% with cancer) showed a non-significant 38% reduction in the relative risk of VTE (RR 0.62, *p* = 0.21) in the tinzaparin arm at the end of the 3–6 month follow-up, which increased to 59% (RR 0.41, *p* = 0.08), becoming significant at 1 year. Tinzaparin appeared to be a valid alternative to VKAs for the therapy of cancer patients with a more favorable benefit-risk ratio, but only at the 1-year follow-up.

It should be noted that much of this evidence dates back to the pre-DOACs era.

### 3.5. Comparison with Other Low-Molecular Weight Heparin

The RIETECAT study [33] compared the long-term efficacy and safety of enoxaparin versus dalteparin or tinzaparin for secondary VTE prevention in adults with active cancer. This multicenter and multinational study used data from the RIETE registry and included 4451 cancer patients who received full doses of the study drugs (3526 enoxaparin; 925 dalteparin or tinzaparin). The results showed a few differences in VTE recurrence (2.0% for enoxaparin vs. 2.5% for dalteparin or tinzaparin) and in the mortality rate (19% vs. 17%). There was a mild increase in major bleeding in the enoxaparin group (3.1% vs. 1.9%). Multivariate analysis and propensity score matching confirmed the absence of statistically significant differences in the risk of VTE recurrence (aHR 0.81; 95% CI 0.48–1.38), major bleeding (aHR 1.40; 95% CI 0.80–2.46), or death (aHR 1.07; 95% CI 0.88–1.30) between the subgroups. The conclusion was that no statistically significant differences were observed about efficacy and safety outcomes over 6 months. Although the percentages of patients with hematological malignancies in RIETECAT were low, this reflects real-life incidence and is similar to the proportion included in clinical trials with LMWHs.

To date, there are no head-to-head RCTs that directly compare tinzaparin with other LMWHs such as enoxaparin or dalteparin. This lack of direct data makes it difficult to determine whether tinzaparin is superior or inferior to other LMWHs in terms of effectiveness in preventing recurrent VTE or in terms of safety, such as the risk of bleeding.

As a result, the comparison between these drugs is primarily based on indirect studies, meta-analyses, and real-world data, which may not be sufficient to establish a clear superiority of one LMWH over another in specific patient populations.

### 3.6. Comparison with Direct Oral Anticoagulants

Cotter et al. [27] in their retrospective observational cohort study, including 61 patients, showed no statistically significant differences between the apixaban and tinzaparin groups in terms of major bleeding events (4.8% vs. 0%, *p* = 0.33) and clinically relevant non-major bleeding events (9.5% vs. 0%, *p* = 0.1). Currently, there are no randomized controlled trials (RCTs) that compare tinzaparin with DOACs. Therefore, the efficacy and safety of tinzaparin compared to DOACs are currently based on limited.

## 4. Tinzaparin in Special Patient Populations

The use of tinzaparin is particularly advantageous in certain subpopulations of cancer patients, thanks to its unique pharmacological characteristics and its well-documented safety profile. In patients with renal impairment (RI), tinzaparin has been shown not to accumulate significantly, even in cases of severe RI (CrCl ≥ 20 mL/min), eliminating the need for dose adjustments. As highlighted by the IRIS [34] and CATCH [20] studies, this feature distinguishes it from VKAs, offering a reduced risk of bleeding. Although considered safe, caution is advised in patients with CrCl below 30 mL/min, where the use of unfractionated heparin (UFH) or LMWH with anti-Xa monitoring may be considered. An additional advantage of tinzaparin is its reversibility with protamine.

For the elderly population, the IRIS [34] and TROPIQUE [9] studies have confirmed a favorable safety profile for tinzaparin. Unlike other LMWHs, no clinically relevant accumulation of anti-Xa activity has been observed, and no significant differences in the risk of major bleeding were found compared to non-elderly patients.

The use of tinzaparin has also been explored in patients with brain tumors, a population at high risk for thrombotic events. The study by Perry et al. [35] showed that tinzaparin can be safely used for primary prophylaxis, reducing the incidence of VTE. However, given the high probability of intracranial bleeding, a careful balance between the antithrombotic benefits and the hemorrhagic risk is essential.

In patients with thrombocytopenia, LMWHs are generally preferred for their more established safety profile. Treatment with tinzaparin in this population requires individualized management, which may include dose adjustments or, in specific cases, a platelet transfusion [36].

Finally, tinzaparin is particularly useful in obese patients, as its dosage is based on actual body weight, without the maximum limitations commonly associated with other LMWHs. This characteristic, supported by data from the RIETE Registry, simplifies clinical management and ensures adequate therapeutic coverage in a population that is often complex to treat [37].

Table 3 summarizes the several categories.

## 5. Tinzaparin in Venous Thromboembolism Prophylaxis for Cancer Patients

Preventing VTE is a crucial aspect of managing cancer patients, both in surgical and non-surgical settings. LMWHs, including tinzaparin, play an essential role in these prophylactic strategies.

### 5.1. Surgical Prophylaxis

All cancer patients undergoing major surgery should be considered for pharmacological prophylaxis, as they have a 2- to 3-fold higher VTE risk compared to non-cancer patients in the perioperative period [38]. ASCO and NCCN guidelines [39,40] recommend continuing anticoagulants for 7–10 days in all patients, extending up to 4 weeks for patients undergoing major abdominal or pelvic surgery for cancer with high-risk features (e.g., reduced mobility, obesity, history of VTE). ESMO guidelines [38] recommend extended prophylaxis for all cancer patients undergoing major abdominal or pelvic surgery.

A Danish study [41] on national registries examined tinzaparin for primary post-surgical prophylaxis in 8645 patients (4273 non-cancer and 4372 with cancer) who underwent major kidney surgery and in 2164 patients (359 non-cancer and 1805 with cancer) who underwent cystectomy. After 6 months, no differences in the rate of VTE events were found in either type of surgery. No VTE-related deaths were recorded.

In a retrospective cohort study by Fioretti et al. [10], 643 patients who underwent surgery for gynecological cancer, who received tinzaparin prophylaxis only during hospitalization, were compared to a cohort of 740 patients who received tinzaparin prophylaxis for up to 4 weeks after the surgery. No differences were found between the two prophylactic strategies, neither for 1-year thrombosis-free survival nor for VTE recurrence rates.

The ESMO guidelines recommend tinzaparin at 4500 anti-Xa IU o.d., starting 12 h after surgery.

Therefore, a speculative result is demonstrated in a prospective cohort study by Quintana et al. [42]. They included 76 colon cancer patients, a prophylactic dose of tinzaparin administered after surgery normalized vascular endothelial growth factor (VEGF) values, whose post-operative increase is responsible for increased tumor growth and metastasis formation.

### 5.2. Non-Surgical/Ambulatory Prophylaxis

Current guidelines generally do not recommend routine pharmacological prophylaxis in ambulatory cancer patients without additional VTE risk factors. However, clinicians should consider anticoagulant prophylaxis in selected high-risk VTE patients receiving chemotherapy, based on a thrombotic risk assessment. Risk assessment models, such as the Khorana Score, have been developed to identify high-risk patients for chemotherapy-associated thrombosis [43].

Despite the emergence of DOACs, LMWHs, including tinzaparin, remain a first-line option in numerous cancer patients, particularly those requiring frequent dose adjustments for chemotherapy-induced thrombocytopenia, those receiving ongoing anticancer therapies with drugs that potentially interact with DOACs, and those at high risk of bleeding (e.g., gastrointestinal or genitourinary cancers) or with brain metastases [43].

## 6. Choice of Anticoagulant Regimen

When choosing between LMWHs like tinzaparin and DOACs for CAT, several patient- and cancer-specific factors come into play.

A key consideration is drug interactions. LMWHs are largely free of significant drug interactions, making them a preferred option for CAT patients taking potent inhibitors or inducers of the cytochrome P450 3A4 (CYP3A4) enzyme and P-glycoprotein, which can affect the pharmacokinetics of direct factor Xa inhibitors [44].

Another factor is the patient’s tolerance for oral medication. Nausea, vomiting, or anorexia can make taking oral drugs difficult, in which case injectable LMWHs are a better choice [34].

Renal function is also critical. Tinzaparin does not accumulate in patients with renal insufficiency, even in severe cases, and does not require dose adjustments. This contrasts with some DOACs, which need dose adjustments or are contraindicated in severe renal insufficiency [20,34].

For patients with specific cancer types, like luminal gastrointestinal or genitourinary tumors, LMWHs are often preferred due to the increased risk of gastrointestinal bleeding associated with DOACs [45].

Platelet count is another important consideration. LMWHs remain the preferred anticoagulant for CAT patients with thrombocytopenia, as there is a lack of data on using DOACs in the presence of severe thrombocytopenia (platelet count < 50 × 10^9^/L) [36].

In patients with brain metastases, LMWHs may be a safer choice to balance the risk of intracranial hemorrhage [35].

Finally, patient preferences are a vital part of shared decision-making. The route of administration, whether it is a daily subcutaneous injection or an oral tablet, and the perceived burden of treatment are important factors to discuss.

International guidelines are evolving to incorporate new data on DOACs but still recognize the fundamental role of LMWHs:

CHEST Guidelines (2021) [46]: recommend oral factor Xa inhibitors (apixaban, edoxaban, rivaroxaban) over LMWHs for the initial and treatment phases of acute VTE in cancer patients (strong recommendation, moderate-certainty evidence). However, they acknowledge that the choice of anticoagulants in CAT is sensitive to patient factors such as drug–drug interactions and tolerance of oral medications. For extended prophylaxis, LMWHs are the preferred alternative to DOACs in CAT patients who cannot receive DOACs.

ESMO Clinical Practice Guidelines (2022) [38]: state that tinzaparin is a treatment option for VTE in cancer patients. Although DOACs represent a new alternative, LMWHs remain a first-line option in numerous cancer patients, particularly those requiring frequent dose adjustments for chemotherapy-induced thrombocytopenia, those receiving ongoing anticancer therapies with drugs that potentially interact with DOACs, and those at high risk of bleeding (such as gastrointestinal or genitourinary cancers) or with brain metastases. For patients with luminal gastrointestinal cancer, LMWHs are preferred.

American Society of Clinical Oncology (ASCO) guidelines [39]: historically, they have recommended LMWHs as first-line therapy for CAT. Although the most recent guidelines include DOACs as an option, LMWHs remain an important choice, especially in specific cases.

International Society on Thrombosis and Haemostasis (ISTH) guidelines [47]: suggests the use of specific DOACs in cancer patients with acute VTE and a low risk of bleeding, after a shared decision with patients. However, LMWHs are still recommended for the management of complex cases, including VTE recurrence despite anticoagulant therapy and thrombocytopenia. Table 4 summarizes the role of tinzaparin base guidelines.

In summary, while DOACs offer advantages in terms of administration convenience, LMWHs like tinzaparin maintain a crucial role, especially in patients with specific characteristics such as renal insufficiency, high risk of gastrointestinal bleeding or complex drug interactions, or when compliance with a once-daily subcutaneous injection is considered more reliable.

## 7. Conclusions

VTE remains a frequent and potentially fatal complication in cancer patients, significantly impacting morbidity, mortality, and the course of anticancer treatments. LMWHs continue to be a cornerstone in the prevention and treatment of CAT. Within this context, tinzaparin emerges as a particularly advantageous option due to its distinct pharmacological properties and well-documented clinical profile, such as for renal safety, in elderly use, and for bleeding profiles.

Tinzaparin offers notable benefits in terms of manageability and safety. Unlike other LMWHs and some DOACs, it does not accumulate in patients with renal insufficiency, a highly relevant feature given the prevalence of renal dysfunction in the oncology population. Tinzaparin has also demonstrated a favorable safety profile in elderly patients.

The efficacy of tinzaparin in preventing recurrent VTE has been confirmed, and a significant reduction in clinically relevant non-major bleeding compared to warfarin further strengthens its role.

Looking ahead, head-to-head comparative studies between DOACs, analyses from real-world registries, cost-effectiveness studies, and targeted research in specific populations such as patients with hematological malignancies and the frail elderly will be necessary to further optimize treatment strategies.

## Figures and Tables

**Table 1:** Different pharmacokinetic profiles among several LMWHs and DOACs [15,16,17,18].

Pharmacokinetc Feature	Tinzaparin	Enoxaparin	Deltaparin	Apixaban	Edoxaban	Rixaroxaban	Dabigatran
Method of administration	Subcutaneous	Subcutaneous	Subcutaneous	Oral	Oral	Oral	Oral
Half-life (hours)	3–4	~4 (single dose)–~7 (multiple doses)	3–5	~12	10–14	5–9 (younger)/11–13 (elderly)	12–14
Bioavailability (%)	86.7	90–91	87	~50	~62	80–100% (10 mg dose); ~66% (20 mg dose on an empty stomach)	~6.5
Clearance	Primarily renal	Primarily renal, with some hepatic metabolism	Primarily renal, with some hepatic and reticuloendothelial metabolism	27% renal; hepatic metabolism (CYP3A4) and intestinal secretion	50% renal; the rest is metabolized and cleared through bile	33% renal (as unchanged drug); 66% hepatic metabolism (CYP3A4) and renal/biliary excretion	80% renal (as unchanged drug)

**Table 2:** Summary of several trials about efficacy of tinzaparin.

Trial Name	Population	Comparator	VTE Recurrence	Bleeding	Mortality
CLOT [19]	672 cancer patients with acute VTE	Warfarin	HR: 0.48 (*p* = 0.002) at 6 months, favoring Dalteparin	No increased risk	Not specified
LITE [20]	200 CAT patients	Warfarin	7% (tinzaparin) vs. 16% (warfarin); RR = 0.44 (*p* = 0.07) at 12 months	27% (tinzaparin) vs. 24% (warfarin); *p* = 0.77	47% in both groups; *p* = 0.54
Main-LITE [21]	200 cancer patients with VTE	Warfarin	7% (tinzaparin) vs. 16% (usual care); *p* = 0.044 at 12 months	27% (tinzaparin) vs. 24% (usual care)	47% in each group
Home-LITE [22]	480 patients with acute DVT (25% with cancer)	Warfarin	3.3% in both groups at 12 weeks and 1 year	Similar in both groups	Similar in both groups
Romera [1]	241 patients with proximal DVT	Acenocoumarol	At 12 months: 5% (tinzaparin) vs. 10.7% (VKA); *p* = 0.11. In cancer patients: 5.5% vs. 21.2%; *p* = 0.06	Not specified	Not specified
CATCH [23]	900 cancer patients	Warfarin	7.2% (tinzaparin) vs. 10.5% (warfarin); *p* = 0.07. In per-protocol analysis: 8.3% vs. 12.7%; *p* = 0.05	Tinzaparin reduced the risk of clinically relevant non-major bleeding (CRNMB)	Not specified
TROPIQUE [9,24]	409 cancer patients with acute symptomatic VTE (Observational study)	None (cohort study)	5.4% at 6 months (*p* = 0.002)	Favorable risk-benefit ratio	Not specified
TiCAT [25]	247 cancer patients (Single-arm trial)	None (single arm)	4.5% in the first 6 months, 1.1% in the 7–12 month period	Low rate of major bleeding	Not specified
USCAT [26]	432 CAT patients (Retrospective, non-interventional study)	None (non-interventional)	5.7% (6–12 months). Higher in specific cancer types.	5.1% (6–12 months). Higher in specific cancer types.	Not specified
Cotter et al. [27]	61 patients	Apixaban	Not specified	No significant difference between groups (*p* = 0.33 for major bleeding and *p* = 0.1 for CRNMB)	Not specified

CRNMB: clinically relevant non-major bleeding; VTE: venous thromboembolism.

**Table 3:** Tinzaparin in special patient populations.

Study	Populations	Advantages of Tinzaparin	Considerations
IRIS study [34] CATCH study [20]	RI	No accumulation even in severe RI, no dose adjustments required (CrCl ≥ 20 mL/min). Lower risk of bleeding compared to AVKs in patients with IR. Protamine reversibility.	Although safe, in severe RI (CrCl < 30 mL/min) UFH or LMWH with anti-Xa monitoring are considered.
IRIS study [34] TROPIQUE study [9]	Elderly	Favorable safety profile. No accumulation of anti-Xa activity.	No significant difference in the risk of major bleeding compared to non-elderly people.
Perry et al. [35]	Brain tumors	Safe primary prophylaxis can reduce the incidence of VTE.	Caution is required, risk-benefit balance between bleeding and thrombosis.
Hsu et al. [36]	Thrombocytopenia	EBPM preferred for broader safety data.	Dose adjustment and/or platelet transfusion required, individualized decisions.
RIETE Registry [37]	Obese patients	Dose calculated on actual body weight, with no maximum limitations for LMWH.	-

CrCl: creatinine clearance; DOACs: direct oral anticoagulants; LMWH: low-molecular weight heparin; RI: renal insufficiency; UHF: unfractionated heparin; VTE: venous thromboembolism.

**Table 4:** The role of tinzaparin based on guidelines.

Guideline	Tinzaparin/LMWH is Preferred in...	Tinzaparin/LMWH is Second-Line/Alternative to...
CHEST (2021) [46]	Patients who cannot receive DOACs for extended prophylaxis. Patients with significant drug–drug interactions or poor tolerance of oral medications.	DOACs for the initial and treatment phases of acute VTE.
ESMO (2022) [38]	Patients requiring frequent dose adjustments (e.g., chemotherapy-induced thrombocytopenia). Patients taking drugs with potential interactions with DOACs. Patients at high risk of bleeding (e.g., gastrointestinal or genitourinary tumors). Patients with brain metastases. (considered a first-line option for numerous patients)	-
ASCO [39]	Specific clinical cases where LMWHs remain an important choice (historically recommended as first-line)	The most recent guidelines include DOACs as an option
ISTH [47]	Complex cases, including VTE recurrence despite anticoagulant therapy. Patients with thrombocytopenia	Specific DOACs for patients with acute VTE and a low bleeding risk

ASCO: American Society of Clinical Oncology; DOACs: direct oral anticoagulants; LMWHs: low-molecular-weight heparins; ISTH: International Society on Thrombosis and Haemostasis; VTE: venous thromboembolism.
